# Preventing Data Ambiguity in Infectious Diseases with Four-Dimensional and Personalized Evaluations

**DOI:** 10.1371/journal.pone.0159001

**Published:** 2016-07-13

**Authors:** Michelle J. Iandiorio, Jeanne M. Fair, Stylianos Chatzipanagiotou, Anastasios Ioannidis, Eleftheria Trikka-Graphakos, Nikoletta Charalampaki, Christina Sereti, George P. Tegos, Almira L. Hoogesteijn, Ariel L. Rivas

**Affiliations:** 1 Department of Internal Medicine, School of Medicine, University of New Mexico, Albuquerque, NM, 87131, United States of America; 2 Los Alamos National Laboratory, Global Security, Mailstop M888, Los Alamos, NM, 87545, United States of America; 3 Department of Biopathology and Clinical Microbiology, Aeginition Hospital, Medical School, National and Kapodistrian University of Athens, Athens, Greece; 4 Department of Nursing, Faculty of Human Movement and Quality of Life Sciences, University of Peloponnese, Sparta, Greece; 5 Department of Clinical Microbiology, "Thriasio" General Hospital, Magoula, Greece; 6 Torrey Pines Institute for Molecular Studies, Port St. Lucie, FL, United States of America; 7 Department of Dermatology, Harvard Medical School, Boston, MA, United States of America; 8 Wellman Center for Photomedicine, Massachusetts General Hospital, Boston MA, United States of America; 9 Human Ecology Department, Centro de Investigaciones Avanzadas, Mérida, México; 10 Center for Global Health-Division of Infectious Diseases, School of Medicine, University of New Mexico, Albuquerque, NM, 87131, United States of America; Food and Drug Administration, UNITED STATES

## Abstract

**Background:**

Diagnostic errors can occur, in infectious diseases, when anti-microbial immune responses involve several temporal scales. When responses span from nanosecond to week and larger temporal scales, any pre-selected temporal scale is likely to miss some (faster or slower) responses. Hoping to prevent diagnostic errors, a pilot study was conducted to evaluate a four-dimensional (4D) method that captures the complexity and dynamics of infectious diseases.

**Methods:**

Leukocyte-microbial-temporal data were explored in canine and human (bacterial and/or viral) infections, with: (i) a non-structured approach, which measures leukocytes or microbes in isolation; and (ii) a structured method that assesses numerous combinations of interacting variables. Four alternatives of the structured method were tested: (i) a noise-reduction oriented version, which generates a single (one data point-wide) line of observations; (ii) a version that measures complex, three-dimensional (3D) data interactions; (iii) a non-numerical version that displays temporal data directionality (arrows that connect pairs of consecutive observations); and (iv) a full 4D (single line-, complexity-, directionality-based) version.

**Results:**

In all studies, the non-structured approach revealed non-interpretable (ambiguous) data: observations numerically similar expressed different biological conditions, such as recovery and lack of recovery from infections. Ambiguity was also found when the data were structured as single lines. In contrast, two or more data subsets were distinguished and ambiguity was avoided when the data were structured as complex, 3D, single lines and, in addition, temporal data directionality was determined. The 4D method detected, even within one day, changes in immune profiles that occurred after antibiotics were prescribed.

**Conclusions:**

Infectious disease data may be ambiguous. Four-dimensional methods may prevent ambiguity, providing earlier, *in vivo*, dynamic, complex, and personalized information that facilitates both diagnostics and selection or evaluation of anti-microbial therapies.

## Introduction

Investigating the properties of infectious disease-related data may improve diagnostics and research. To that end, four-dimensional (three-dimensional plus temporal) approaches may be useful. While pursued for many years [1], four-dimensional (4D) methods remain scarce: the *Web of Science*^™^ currently retrieves less than ten hits when ‘four-dimensional analysis’ and ‘infection’ are searched.

Biological *complexity* is a major set of properties to be investigated. Infectious disease data may reveal, at least, four properties associated with complexity: (i) *emergence*, (ii) *irreducibility*, (iii) *unpredictability*, and (iv) *autonomy* [2–8]. *Emergence* is the central concept: it refers to the *new* features detected when a complex structure is assembled, which are not observed when its constitutive parts are individually measured [2]. *Emergence* cannot be reduced to the properties of any one variable. *Unpredictability* denotes the inability to anticipate emergence when only ‘simple’ and/or isolated variables are analyzed, e.g., immunoglobulins express emergent properties, which are neither reducible to first principles nor predictable [3]. Similarly, the emergent features of three-dimensional (3D) interactions–e, g, those associated with multi-cellularity–cannot be predicted by bi-dimensional models [4]. Autonomy is characterized by *non-linearity*: because causes and effects are not coupled, the effect (*emergence*) is numerically autonomous from the cause(s) [2].

*Emergence* may reflect *hidden relationships*: information usually non-observable may become apparent when the data are shaped as complex structures [9]. While complexity has been partially investigated in infections affecting non-human species [9, 10], this group of properties has not yet been explored in humans, including personalized medical practices.

A second set of interesting properties includes *‘one-to-many’/‘many-to-one’ relationships* [11, 12]. Such properties occur when one structure (e.g., a cell type) participates in two or more functions and also when several structures act in the same function, e.g. (i) monocytes both promote and destroy neutrophils (‘one-to-many’ interactions) and (ii) both lymphocytes and monocytes are involved in antigen recognition (‘many-to-one’ interactions [13, 14]).

*Spatial-temporal relativity* is another property of biological data, not yet assessed in infectious diseases [15]. It refers to data collected over long periods of time, which may occupy a small portion of the space (plot) used to analyze the data, while observations collected over short periods of time–such as recent infections–may occupy a large plot space.

Biological *spatial-temporal relativity* may result in non-interpretable (ambiguous) data. *Ambiguity* occurs when numerically *similar* data express *different* biological conditions [16].

To prevent ambiguity, *dynamics* (temporal changes) should be investigated. To assess dynamics, it is necessary to address the fact that, in infections, *numerous temporal scales may co-exist*. Because anti-microbial immunity may simultaneously involve responses lasting from nanosecond to weeks and larger temporal scales [17, 18], any pre-selected temporal scale may fail to capture all biological changes–at least some processes will be missed.

To avoid information loss, *pattern recognition* should be considered. When arrows that connect two temporal observations are used (temporal data *directionality*) and 3D/4D patterns are assessed, emergence may be detected, even when space is not uniform [19–24].

Pattern recognition of *perpendicular data subsets* is facilitated by the use of *3D/4D* plots. Perpendicular data subsets reflect *non-overlapping data distributions*. Such data subsets tend to differ at statistical levels, regardless of their number of observations.

Methods that detect 3D perpendicular data subsets could address the limitations of the ‘single structure/single sequence/single function’ paradigm–which no longer holds [25]. Because biology is characterized by *much fewer structures than functions*–as shown by the fact that only five cell types (lymphocytes, monocytes, neutrophils, eosinophils, and basophils) protect against a much larger number of microbes–, methods that explore data combinations are needed, which may take advantage of (i) a rather low number of *interdependent* relationships [26], (ii)‘one-to-many/many-to one’ relationships (e.g., the fact that no cell type, alone, performs any function, but two or more cell types do [12, 13]); and (iii) the informative value of emergence. Validity augments when hidden information is unveiled [9, 27, 28].

To validate methods likely influenced by the unpredictability of biological complexity, numerous comparisons–across individuals, populations, host species and/or microbes–are crucial. When similar patterns are observed across species and pathogens, the likely explanation is that such patterns are highly conserved and, therefore, reproducible [29–31].

Here, infectious disease data were investigated with two methods: (i) an approach that assesses cell types in isolation; and (ii) a method that measures *immuno-microbial-spatial-temporal* data *interactions*. The goals of this pilot study were (i) to elucidate whether infectious disease data express ambiguity; and (ii) to determine whether methods that capture complex dynamics prevent ambiguity and/or extract more information.

## Materials and Methods

### Longitudinal canine and human leukocyte and microbial data

Three sets of data on human infections were analyzed. The first set included seven septicpatients with no history of chronic non-infectious diseases, who were infected by various bacteria and met at least three systemic inflammatory response syndrome (SIRS) criteria [32]: body temperature > 38°C, heart rate > 90 beats/minute, tachypnea or hyperventilation (>20 breaths/minute or P_ACO2_ < 32 mm Hg at sea level), and white blood cell count ≥ 12000 or ≤ 4000/μl. Blood leukocyte percentagess were determined, over three days, since hospital admission (Table A in S1 File).

Human blood leukocytes were also collected from two non-septic but infected patients (Tables B, C in S1 File). The first case was a 49-year old man previously diagnosed with human immunodeficiency virus (HIV), who presented with ~ 1% CD4+ T cells and, over four months, experienced methicillin-resistant and -sensitive *Staphylococcus aureus* (MRSA and MSSA, respectively) mediated infections. The second case was a 60-year old man that received a hip implant who, over seven months, had recurrent MSSA infections [33, 34].

To elucidate whether the 4D method could be applied to non-human species, blood leukocytes and bacteriological tests were explored in one dog (Table D in S1 File). Over 9 months, the animal was spontaneously infected, first, by the opportunistic *Enterobacter cloacae* [35] and, later, by *Staphylococcus pseudointermedius* (a common cause of skin infections [36]).

### Laboratory methods

Identification and quantification of human leukocytes (lymphocytes [L], neutrophils [N] and monocytes [M]) were conducted with an automated hematology analyzer (Coulter LH 780 Analyzer, Beckman Coulter International SA, Nyon, Switzerland). Blood culture was performed with the automated Bactec 9249 instrument (Becton Dickinson, New Jersey, USA). The pathogens isolated from blood were identified and tested for their antimicrobial susceptibility with the automated microbiology system Phoenix 100 (Becton Dickinson, New Jersey, USA). Similar techniques were utilized to conduct canine studies in a veterinary hospital.

### Methods

These studies were approved by the Scientific Committee of the Thriasio Hospital, Magoula, Greece (protocol 57/16-02-2015) and the Human Research Review and Institutional Animal Care and Use (IACUC) committees of the University of New Mexico (protocols numbers 13–463 and 13-101022-T-HSC). Informed consent was not provided because this study was conceived after patients were discharged or died. Patient records were de-identified prior to analysis.

To diminish data variability, a *single* (one data-point wide) *line of observations* was created–a structure that eliminates variability from all dimensions except the one defined by the single line. For example, a bi-dimensional plot that measures, in one axis, the phagocyte/ lymphocyte ratio (the sum of M and N percents divided over the L percentage) and, in the other axis, the L%, creates a single line of data points [37].

*Three-dimensional* (3D) plots were used to extract more information. The addition of depth, to height and width, may uncover data patterns that uni- or bi-dimensional plots cannot reveal.

To estimate *complexity*, *dimensionless indicators* were utilized [9, 10, 38, 39], consisting of *combinations* of counts, percentages, ratios, or products derived from primary variables. When the percents of L, M, and N are used in an equation that includes many interactions (e.g., the {[M/L * N/M] / [N/L * L/M]) over ([M+L/N] * [L+N/M] / [N+M]/L * [M/N]}), a dimensionless number is created: the number produced does not describe any known biological structure.

Dimensionless indicators (DI) can capture many levels of *complexity*. For instance, in the DI described above, one level of complexity (level I) is estimated by each ratio of the first element or ‘numerator’ (M/L, N/M, N/L, and L/M). Two more interactions (of level II complexity) are measured by each product (M/L * N/M, N/L * L/M). Complexity level III is evaluated by the composite ratio of the numerator ([M/L * N/M] / [N/L * L/M]). Because the second element (‘denominator’) has the same structure, the number of interactions doubles when the denominator is calculated. An additional interaction (complexity level IV) is generated when the numerator and the denominator are simultaneously analyzed. When three DIs are assessed in 3D space, the number of interactions increases three times and one more interaction (level V complexity) is produced when the overall (3D) relationship is plotted. Thus, the example shown above covers at least (4 x 2 +1 x 2 +1 x 3 +1) *58 interactions* and *five levels of complexity*.

Furthermore, a sixth level of complexity was considered when *dynamics* were addressed [18]. Using non-numerical indicators (arrows that connected pairs of consecutive observations), *temporal directionality* was investigated. Such arrows detected multi-directional data flows.

This design revealed distinct 3D/4D patterns, which distinguished data subsets. The validity of each subset was determined by analyzing microbial test results and/or leukocyte data.

### Data analysis

Because all DIs–except a few indicators (N/L, M/N, M/L, N*L, M*N and M*L)–, included the same data contents, DI were not described but identified with descriptors written in italics (e. g., *AAR*). Because DIs expressed hypothetical interactions, they were not biologically observable and, statistically, they were neither interpretable nor predictable [40]. However, after distinct spatial patterns (such as perpendicular data inflections) were detected, data subsets were differentiated and statistical comparisons among subsets were justified. Because differences among data subsets were based on their immune profiles, validations were biologically grounded. Dimensionless indicators were generated by a proprietary algorithm [9]. Leukocyte percents, products, or ratios were compared with the Mann-Whitney test, which tested whether medians differed across data subsets (Tables E-H in S1 File). Plots and statistical analyses were created or conducted with Minitab 17^®^ (State College, PA, USA). The data described in the Table I in S1 File can be analyzed statistically with the procedure reported in the footnote of Table J in S1 File.

## Results

**Fig 1:**
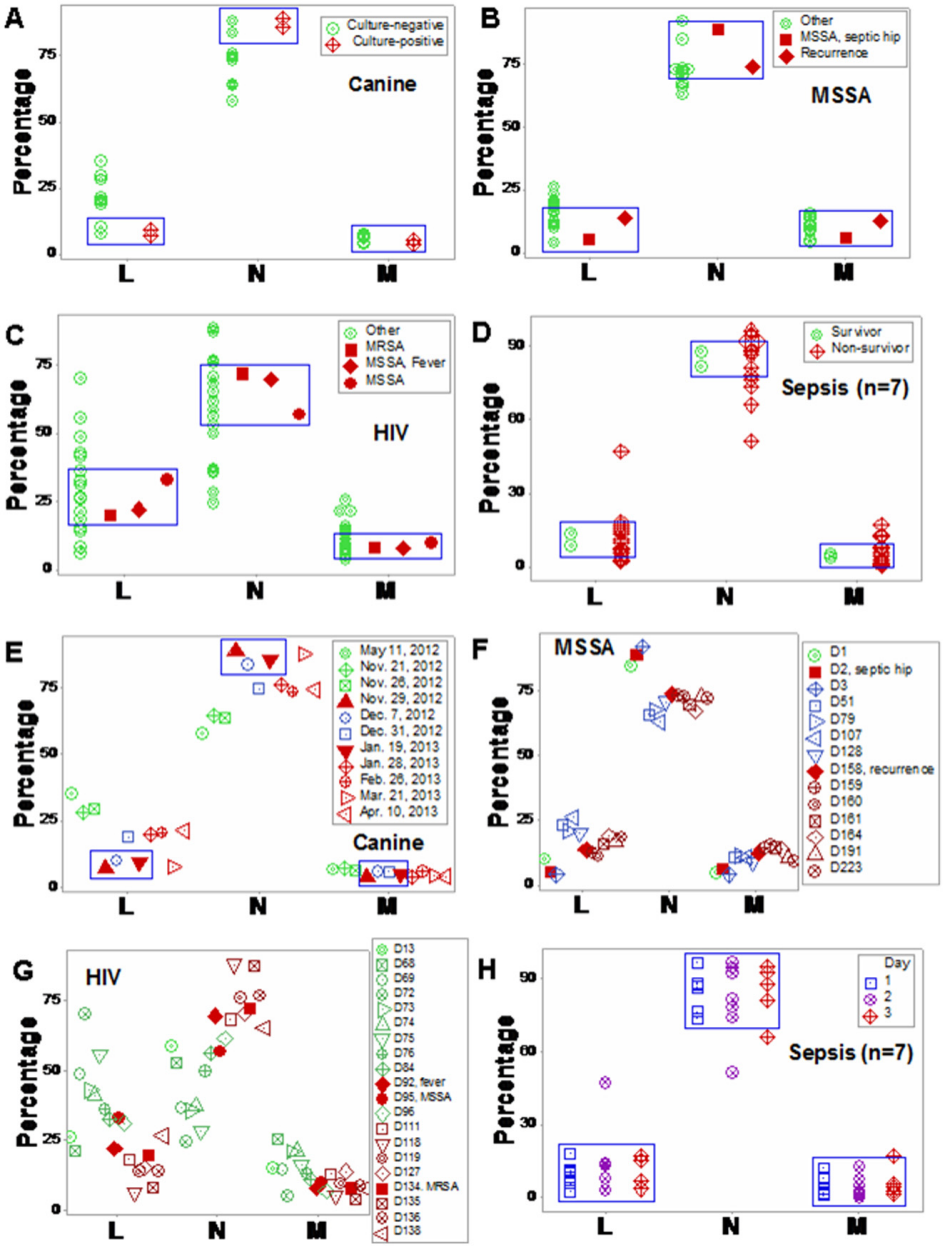
Classic analysis of immuno-microbial data. The classic method did not discriminate: leukocyte data distributions overlapped among different biological conditions, such as fever-positive and fever-negative individuals or individuals that recovered or did not recover from infections (blue boxes, **a-d**). The analysis of temporal data did not improve discrimination (**e-h**). Four studies were evaluated, including: (i) one dog [**a, e**], (ii) one human infected by MSSA [**b, f**]; (iii) one human HIV case, with a secondary MRSA infection [**c, g**]), and (iv) seven humans presenting with sepsis [**d, h**]).

The classic method was not predictive: leukocyte data did not separate different clinical presentations, such as infected and non-infected or fever-positive and -negative individuals (blue boxes, Fig 1a–1d). The analysis of temporal data did not improve discrimination (Fig 1e–1h).

**Fig 2:**
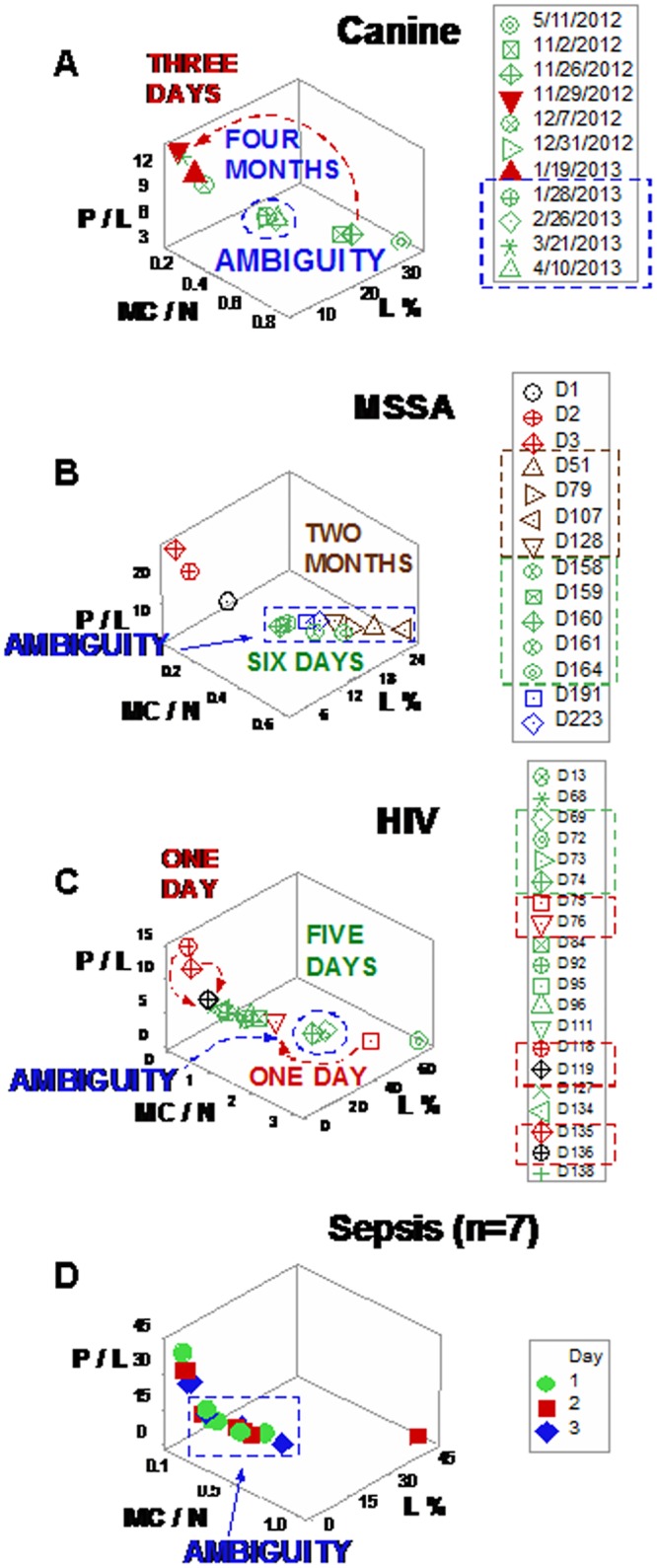
Spatial-temporal data ambiguity. Ambiguity (numerically similar observations that expressed different biological conditions) was also documented when three-dimensional (3D) relationships were explored and single (one data point-wide) lines of observations were utilized to explore longitudinal data. *Ambiguity* exhibited *spatial-temporal relativity*: data points that corresponded to recent infections occupied more space and/or exhibited broader data ranges than observations not associated with recent infections and/or recorded over longer periods (**a-d**). For instance, observations recorded within three days (red arrow, **a**) displayed a broader data range than observations collected over the following four months (blue oval, **a**). Consequently, no numerical value of leukocyte data, per se, could distinguish recent from older or protracted responses.

**Fig 3:**
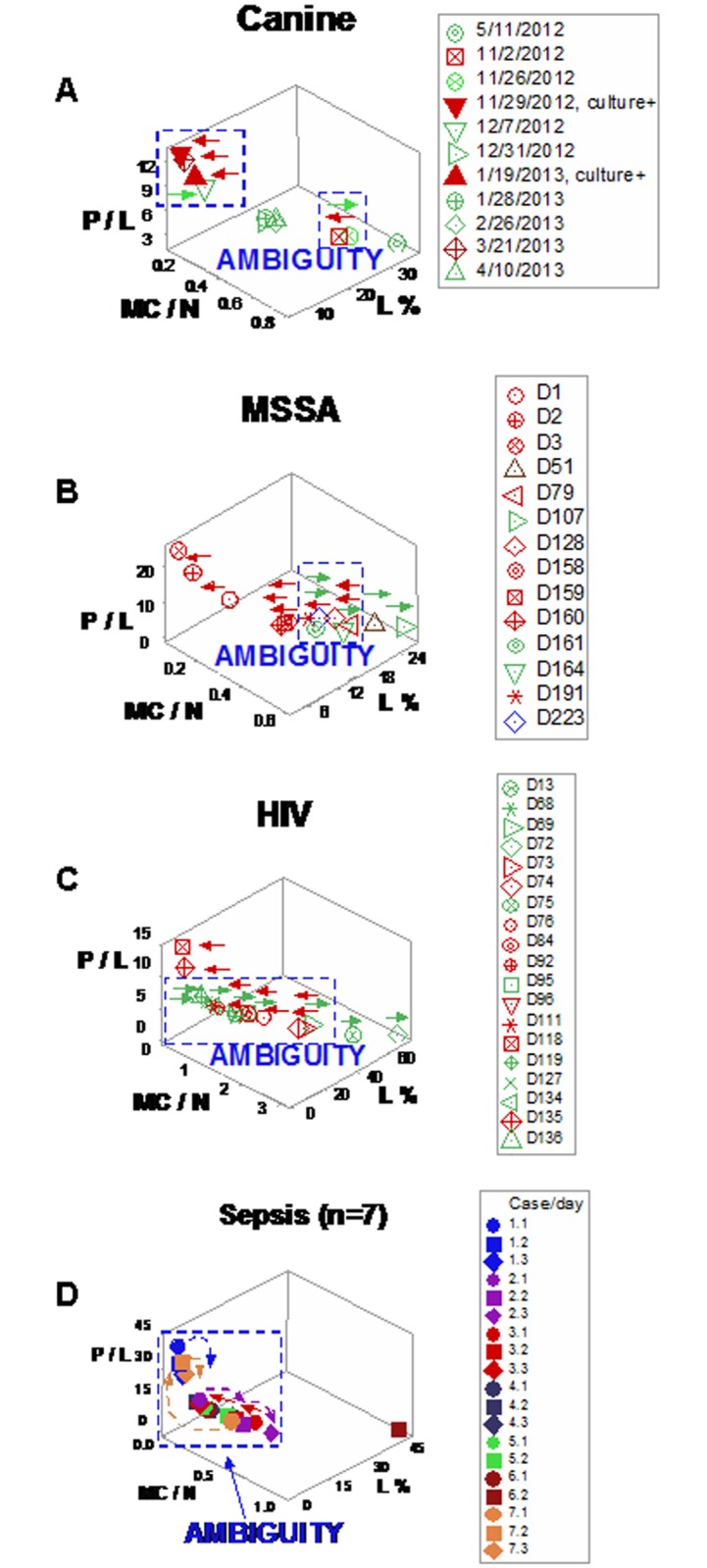
Multi-directional data ambiguity. Ambiguity was also expressed when temporal data directionality was evaluated: arrows that connected pairs of consecutive observations displayed different temporal directionality even when they exhibited similar numerical information (boxes, **a-d**). Such pattern indicated that some dynamic changes took place at temporal scales smaller than the one utilized. Therefore, the 3D, single line of data points defined by the L%, the phagocyte/lymphocyte (P/L) and the mononuclear cell/neutrophil (MC/N) ratios failed to discriminate dynamics: some observations with similar numerical values, which expressed different biological conditions, were not distinguished.

Lack of discrimination was also observed when single (one data point-wide) lines were investigated within three-dimensional (3D) plots. In all studies, some *numerically similar* data points expressed *different clinical* conditions (*ambiguity*). Ambiguity was associated with *spatial-temporal relativity*: data points that corresponded to recent infections occupied more space and/or exhibited broader data ranges than observations not associated with recent infections and/or recorded over longer periods (Fig 2a–2d). *Multi-directional temporal ambiguity* was also detected: some pairs of consecutive observations displayed similar numerical values but expressed different temporal directionality (arrows within boxes, Fig 3a–3d).

**Fig 4:**
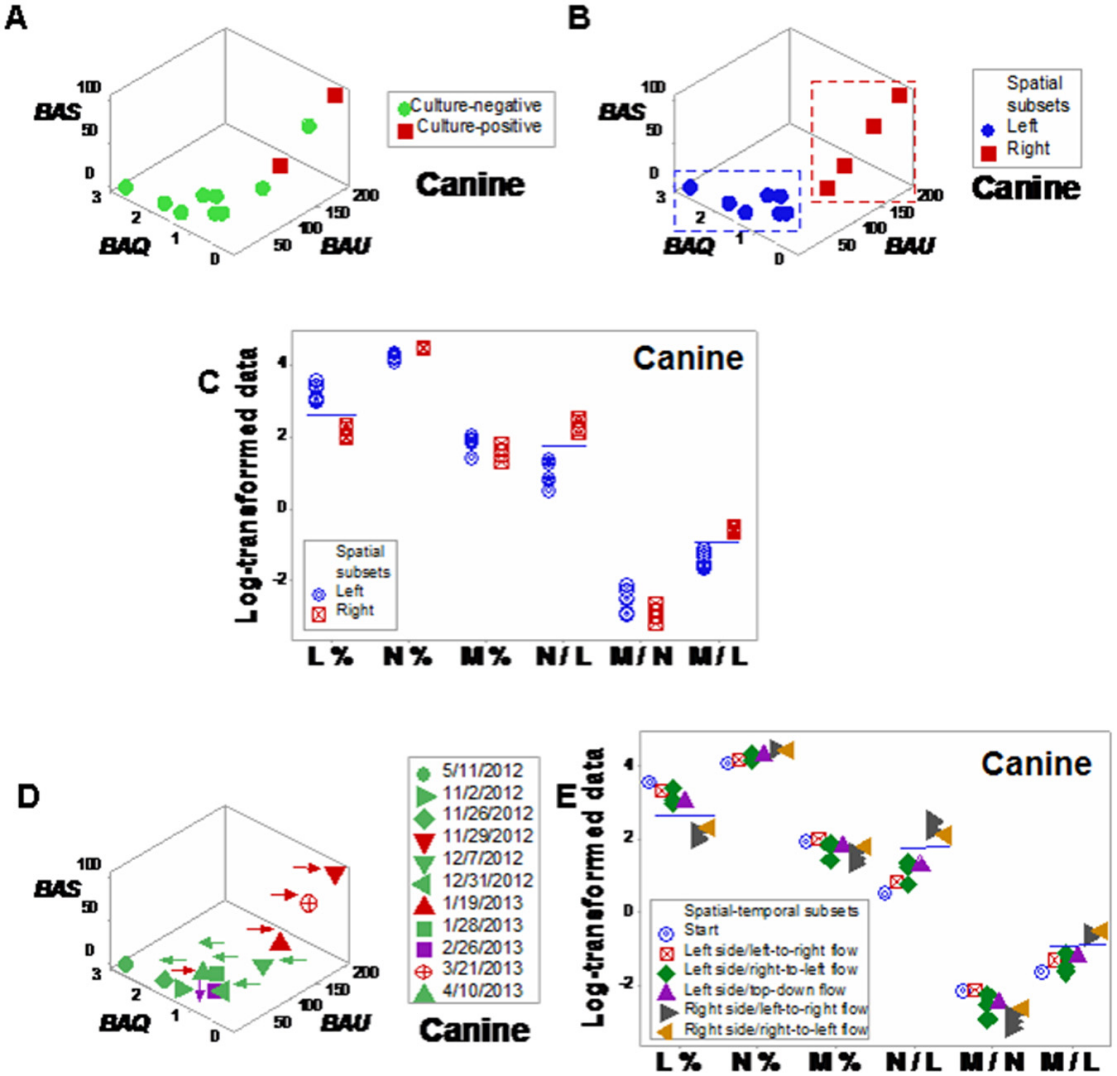
Canine leukocyte spatial-temporal relationships. When dimensionless indicators (DIs) were utilized and three-dimensional (3D) patterns were considered, canine data revealed two (‘left’ and ‘right’) subsets (**a, b**). Spatial data subsets exhibited non-overlapping lymphocyte percentages and N/L and M/L ratios (**c**). When temporal data directionality was considered, arrows expressing different directionality (**d**) increased discrimination: 4D (spatial-temporal) patterns distinguished five subsets (in addition to the first observation) and non-overlapping N% differentiated the ‘right side/left-to-right flow’ observations from the first one (horizontal lines indicate non-overlapping data subsets, **e**).

Discrimination improved when dimensionless indicators (DIs) were utilized. For instance, three-dimensional (3D) canine patterns revealed two (‘left’ and ‘right’) data subsets (Fig 4a and 4b). The L%, the N/L and M/L ratios of such data subsets did not overlap (Fig 4c).

Arrows with different directionality helped distinguish observations that expressed similar numerical values. Three data flows were detected in canine responses: (i) a left-to-right flow (red arrows), (ii) a right-to-left flow (green arrows), and (iii) a vertical, top-down flow (purple arrow, Fig 4d). Spatial-temporal flows differentiated five subsets from the first data point. Non-overlapping N/L and M/L ratio values discriminated left-side/right-to-left flow- from left-side/top-down flow-related observations (horizontal lines, Fig 4e). In addition, non-overlapping M/N data intervals distinguished right-side/left-to-right- from right-side/right-to-left flow data points (Fig 4e). When leukocyte profiles were analyzed, at least four comparisons among spatial (3D) or spatial-temporal (4D) subsets reached statistical significance (Table E in S1 File).

**Fig 5:**
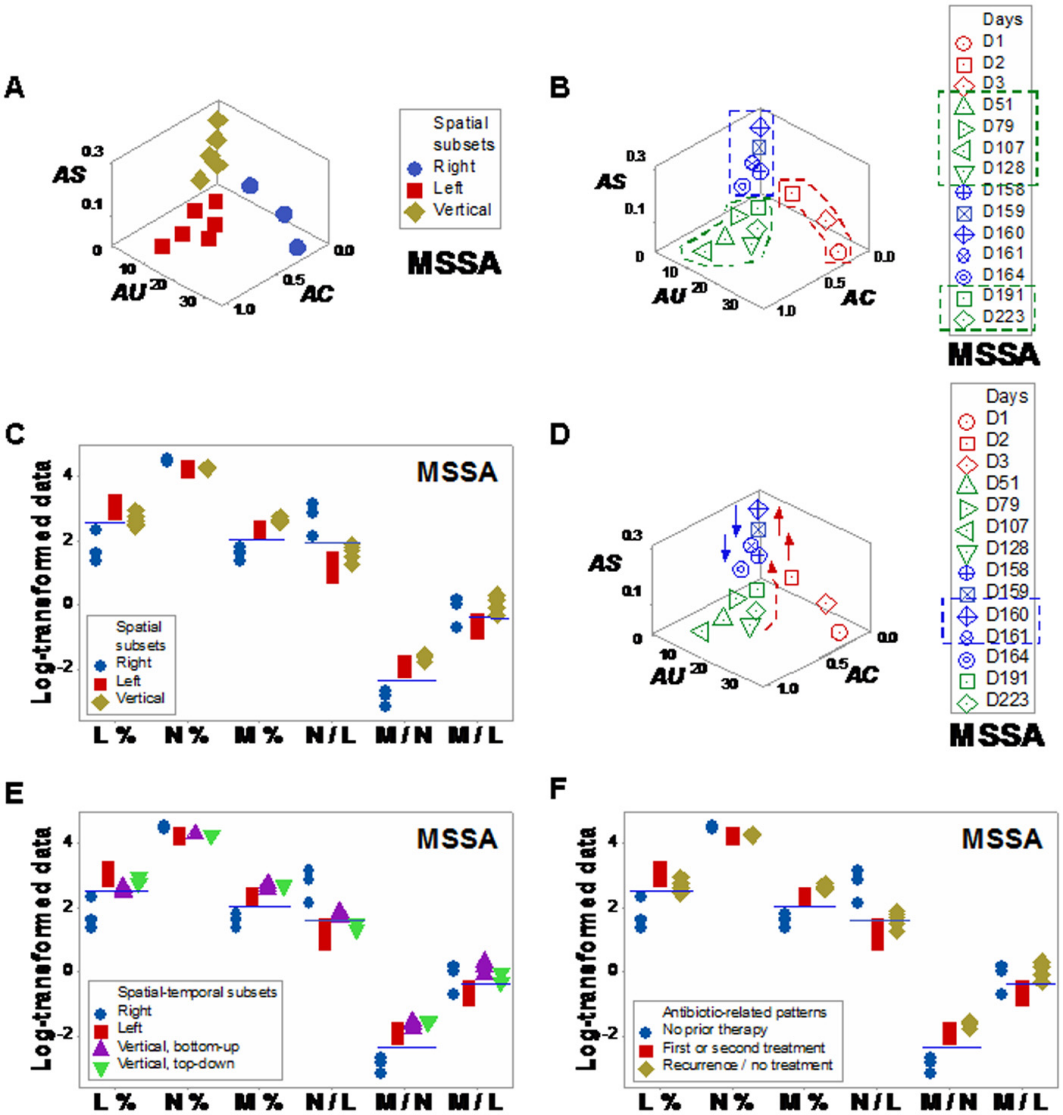
Human leukocyte spatial-temporal (MSSA/hip implant-related) relationships. Three data subsets were identified when the MSSA/hip implant human case was explored with dimensionless indicators (**a**). All data points associated with antibiotic therapy were clustered within one subset (green polygon, **b**), even though antibiotics were administered in two non-consecutive periods (green boxes, **b**). The ‘vertical’ subset exhibited statistically significantly higher M/L values than the ‘bottom, left’ subset (**c**). When arrows that connected pairs of consecutive observations were assessed, three ‘bottom-up’ and two ‘top-down’ observations were detected (red and blue arrows, respectively, **d**). Changes in directionality were detected within one day: at days 159/160, one ‘bottom-up’ data point was followed by one ‘top-down’ observation (**d**). Spatial-temporal patterns (temporal data flows) differentiated four data subsets (**e**). The use of arrows distinguished ‘vertical, bottom-up’ from ‘vertical, top-down’ observations (horizontal line, **e**).

Three spatial subsets were identified when the MSSA hip implant human case was explored with dimensionless indicators (Fig 5a). All data points associated with antibiotic therapy were clustered within one subset, even though antibiotics were administered in two non-consecutive periods (green symbols and green boxes, Fig 5b). Fifteen leukocyte-related comparisons differed among the three spatial patterns (*P*≤0.04, Fig 5c and Table F in S1 File). Arrows that connected pairs of consecutive observations detected three ‘bottom-up’ and two ‘top-down’ observations (Fig 5d, red and blue arrows, respectively). Four spatial-temporal patterns were detected (Fig 5e). ‘Vertical/bottom-up’ observations showed statistically significantly higher N/L values than ‘vertical/top-down’ data points (horizontal line, Fig 5e and Table F in S1 File). The MSSA case also indicated antibiotic-related effects. Higher M/L values were observed after the first, but before the second antibiotic treatments (horizontal line, Fig 5f).

**Fig 6:**
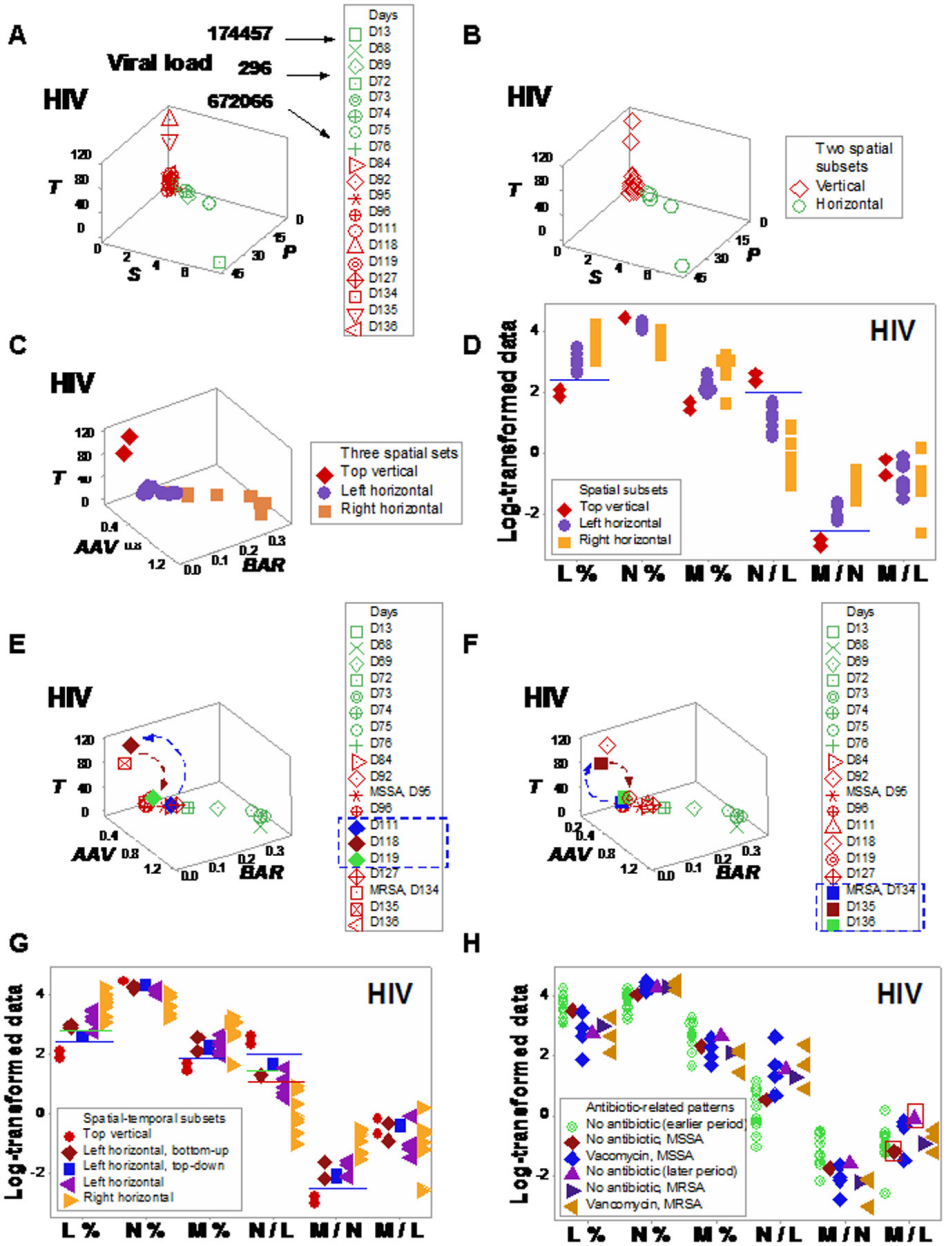
Human leukocyte spatial-temporal (HIV/MRSA-related) relationships. Viral load values of the HIV+ patient were not informative: they exhibited more than 1000-fold changes among clinically stable observations (arrows indicating green symbols, **a**). In contrast, dimensionless indicators (DIs) differentiated two spatial (‘vertical’ and ‘horizontal’) subsets, which included two MRSA isolations within the vertical subset (set I, **b**), while all bacteria-negative data points were horizontally located (set II, **b**). A second set of DIs separated the ‘vertical’ data points into two sub-subsets: (i) the ‘top vertical’ and (ii) the ‘left horizontal’ groups, which did not overlap with the remaining (‘right horizontal’) data points (**c**). At least the L% and the M/N ratio distinguished the three spatial data subsets (**d**). More information was extracted when arrows that connected pairs of consecutive observations were measured (**e, f**). The assessment of *spatial*-*temporal data directionality* differentiated, twice, changes that took place within one day (days 118–119; and 135–136; arrows, **e, f**). While the spatial (3D) analysis detected only two or three data subsets (**b, c**), the spatial-temporal (4D) assessment distinguished five data subsets (**g**). For instance, the L%, M%, N/L, and M/N ratios differentiated ‘top vertical’ from the remaining observations (blue horizontal lines, **g**). The L% and N/L ratio also distinguished the ‘left/top-down’ observation from the ‘left/bottom-up’ observations (green horizontal lines, **g**). Furthermore, the N/L ratio discriminated the ‘right horizontal’ from the remaining subsets (red horizontal line, **g**). Some leukocyte profiles were associated with antibiotic therapy, for instance, higher M/L values were observed after antibiotics were prescribed, even after antibiotic therapy was discontinued (**h**).

Informative patterns were also observed, in the HIV case, when DIs were analyzed. While viral load values were not informative (they exhibited more than 1000-fold changes among clinically stable observations, Fig 6a), data associated with bacterial isolations predominated in the ‘vertical’ data subset (Fig 6b). A second set of DIs amplified the detection of observations horizontally displayed (Fig 6c). Combining the patterns displayed by the first set of DIs with those expressed by the second set, data points were divided into ‘top vertical’ and ‘left horizontal’ observations and, together with ‘right horizontal’ data points, three data subsets were spatially differentiated (Fig 6d). The immune profile of such datasets showed non-overlapping L% and M/N ratio intervals (Fig 6d). *Temporal flows* distinguished data points that displayed similar numerical values but differed in directionality (days 118–119; and 135–136; arrows, boxes, Fig 6e and 6f). While the spatial (3D) analysis detected three data subsets, the spatial-temporal (4D) assessment differentiated five subsets and exhibited non-overlapping L %, M %, M/N, and N/L distributions (Fig 6g). Seven comparisons among subsets detected by either 3D or 4D patterns reached statistically significant differences (Table G in S1 File).

Long-term antibiotic-related immune responses were also suggested in the HIV case. Higher M/L values were observed, even after cessation of antibiotic therapy (boxes, Fig 6h).

**Fig 7:**
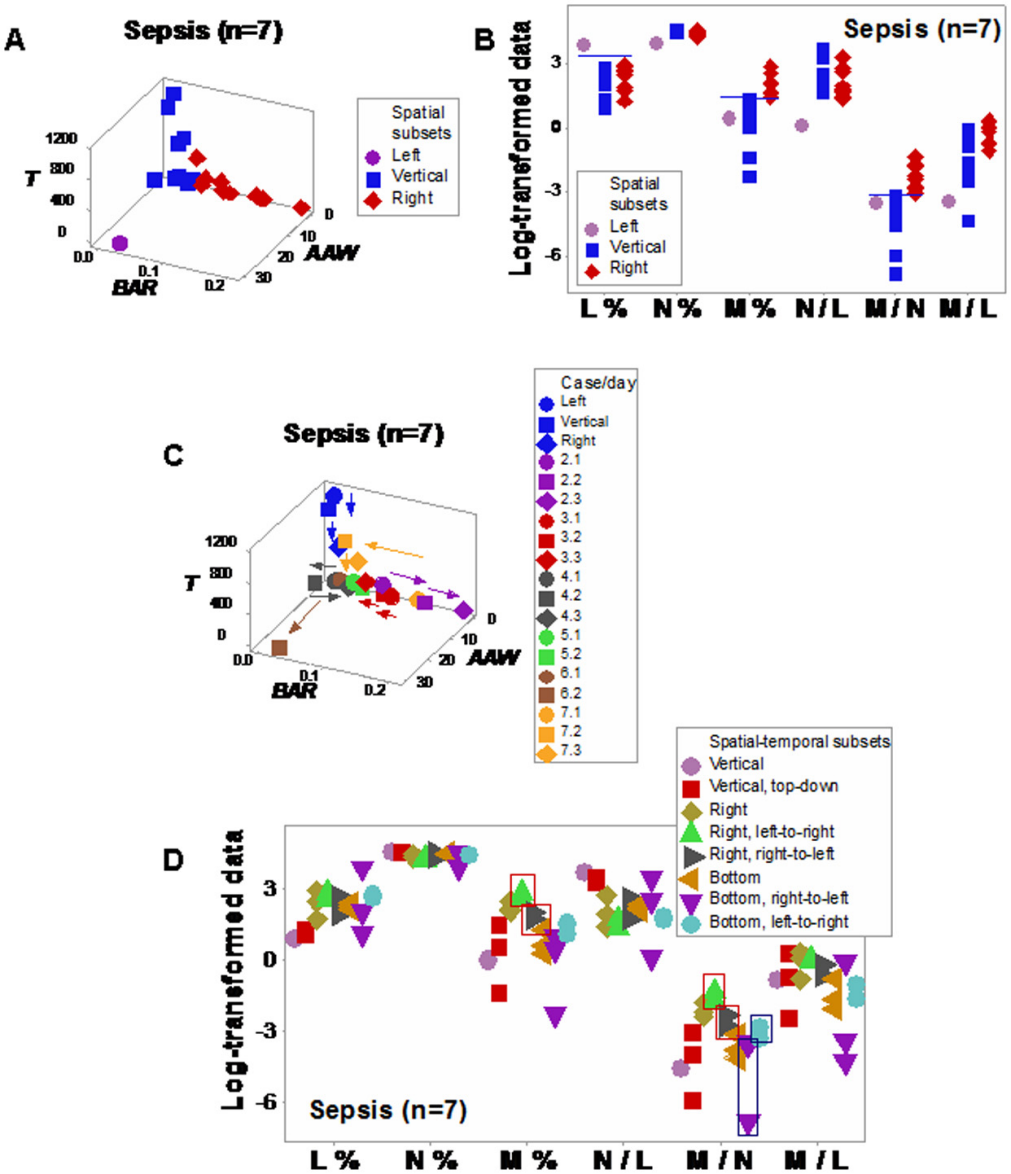
Longitudinal relationships in septic humans. Spatial patterns differentiated three data subsets among 7 septic patients analyzed with dimensionless indicators: (i) a vertical subset, (ii) a right subset, and (iii) the remaining observation, or ‘left’ subset (**a**). Higher M% and M/N ratio values distinguished the ‘right’ subset from the remaining data points, while higher L% and lower N/L ratio values differentiated the ‘left’ data point from the remaining observations (horizontal lines, **b**). Discrimination further improved when temporal and multidirectional data flows were assessed: several numerically similar observations displayed different directionalities (**c**). While not all observations could be analyzed statistically because some patterns included only one or two data point(s), the spatial-temporal analysis detected non-overlapping M% and M/N ratio distributions that differentiated by the ‘right’ subset with a left-to-right directional flow from the ‘right’ subset with a right-to-left flow (boxes, **d**). Non-numerical information (arrows) also distinguished ‘bottom/right-to-left’ from ‘bottom/left-to-right’ observations (boxes, **d**).

When dimensionless indicators were used to evaluate septic patients, three (‘left’, ‘vertical’, and ‘right’) subsets were detected (Fig 7a). The L% and the N/L ratio differentiated the ‘left’ from the remaining subsets, while M% values distinguished the ‘vertical’ from the ‘right’ subset (Fig 7b). Different temporal data flows were exhibited by several data points that expressed similar numerical values, which discriminated eight spatial-temporal patterns (Fig 7c and 7d). While several of such subsets only included one or two data point(s) (so, no statistical test could be conducted), spatial-temporal patterns prevented ambiguity; for instance, ‘right side/left-to-right’ and ‘bottom/right-to-left’ observations were differentiated from ‘right side/right-to-left’ and ‘bottom/left-to-right’ data points, respectively (Fig 7d).

**Fig 8:**
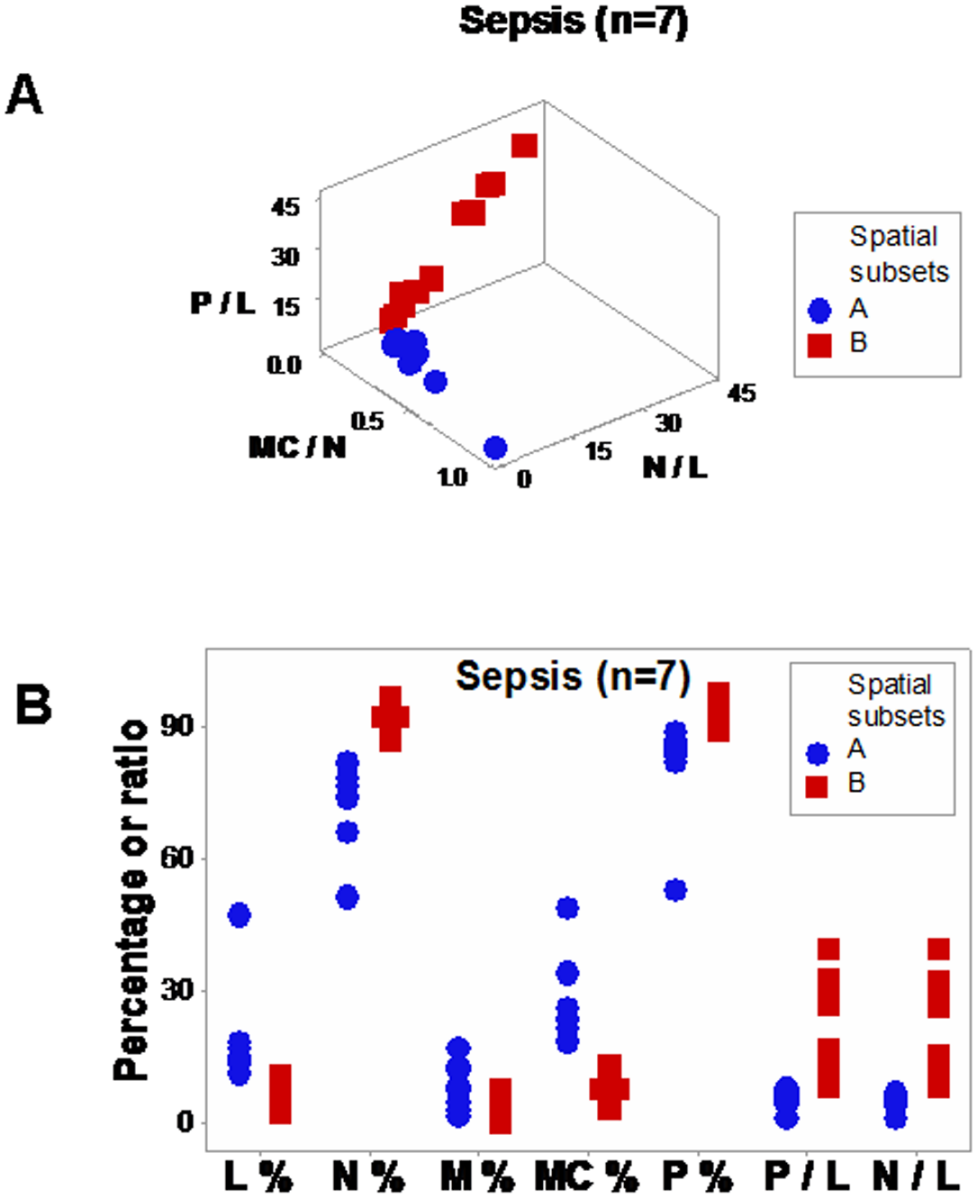
Spatial analysis of low-complexity indicators. Even in its simplest version–which did not utilize dimensionless indicators–, the 4D method was more informative than the non-structured analysis reported in Fig 1. When low-complexity indicators that measured interactions involving two or more cell types were spatially analyzed (the phagocyte/lymphocyte [P/L], the mononuclear cell/neutrophil [MC/N], and the neutrophil/lymphocyte [N/L] ratios), two subsets of septic patients-related data, perpendicular to one another, were detected (**a**). The spatial analysis exhibited a single (one data point-wide) line of observations (**a**). When leukocyte data were partitioned according to the spatial patterns, several comparisons reached statistical significance ((**b)** and Table J in S1 File).

Discrimination was also achieved when leukocyte-explicit, low-complexity–not dimensionless–indicators were utilized. Two non-randomly distributed (perpendicular) data subsets were detected when the phagocyte/lymphocyte (P/L), the mononuclear cell/neutrophil (MC/N), and the neutrophil/lymphocyte (N/L) ratios were evaluated in septic patients (Fig 8a). When observations were classified according to the observed spatial patterns, eight between-subset comparisons differed statistically (Fig 8b; Tables I, J in S1 File).

**Fig 9:**
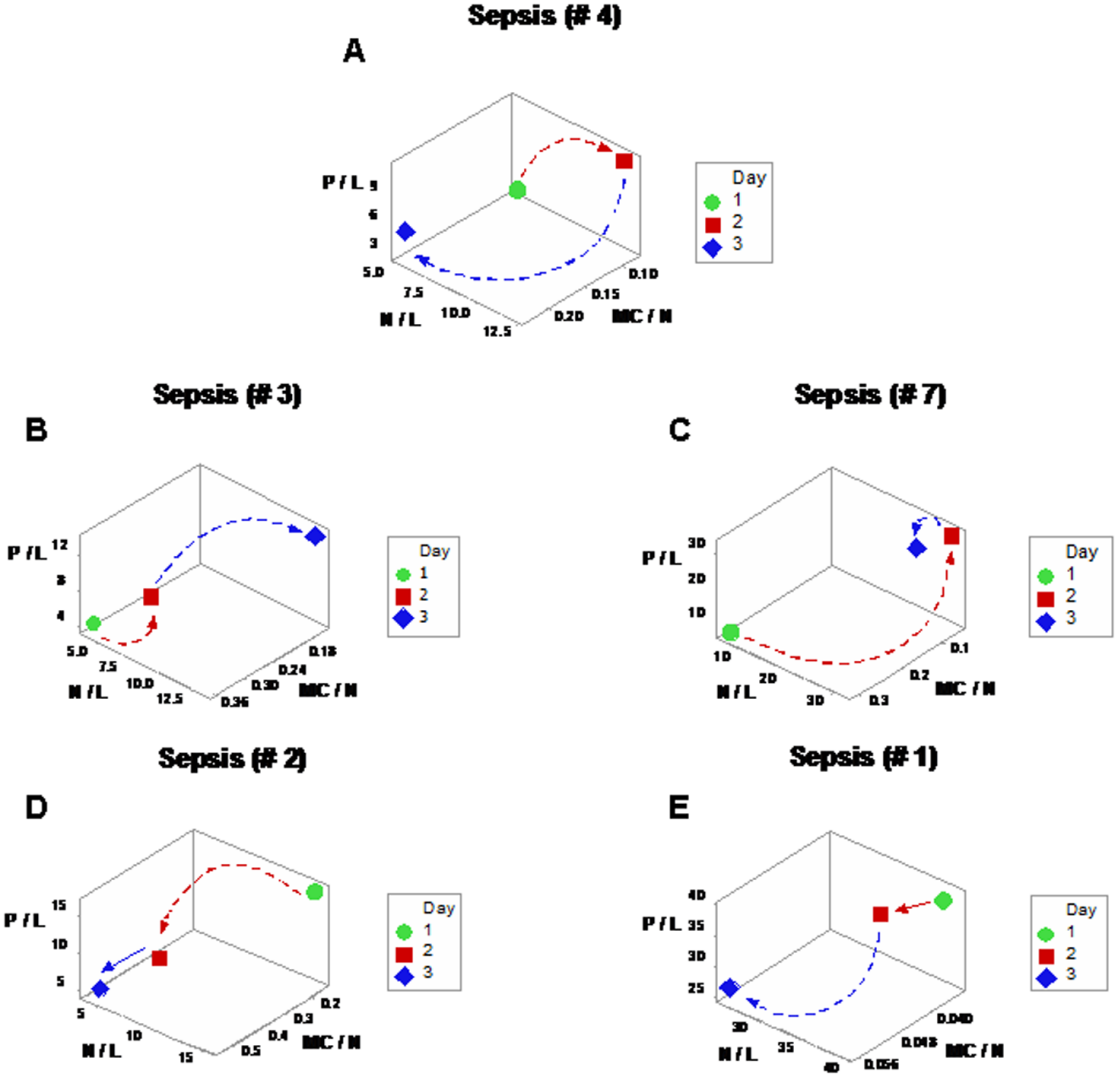
Spatial-temporal and personalized data analysis. When the leukocyte data of five septic patients tested daily over three days were analyzed on personalized bases, several *temporal patterns* were observed (the data of the two remaining septic patients were not analyzed because they were tested only two days). At least *two directionalities* were differentiated: (i) data flows that came from the center or left and, over time, moved to the right (‘from left-to-right’, **a, b**); and (ii) responses that followed the opposite directionality (**c-e)**. These responses were induced by: *A*. *baumannii* (**a**), *E*. *faecalis* (**b**), *S*. *liquefaciens* (**c**), and *E*. *coli* (**d, e**).

Spatial-temporal information also supported personalized assessments, even when low-complexity interactions were measured. Based on data directionality, flows that started in the center or left and, over time, moved to the right (Fig 9a and 9b) were distinguished from those that progressed from the right to the left side of the plot (‘right-to-left’ flows, Fig 9c–9e).

## Discussion and Conclusions

Data ambiguity is not rare: it is observed across species and syndromes, when infectious disease-related data are analyzed as simple (non-structured) variables. To prevent ambiguity-related errors, spatial-temporal (4D) data interactions were evaluated. Findings revealed that 4D analysis may distinguish data subsets and prevent ambiguity.

### Methodological considerations

The detection of *hidden data interactions* (i.e., emergent patterns) demonstrated that 4D approaches can extract more information than alternatives. Because validity is questionable when hidden information is not ruled out, findings supported the validity of the combinatorial approach [41, 42].

In addition, *spatial-temporal relativity* was documented [15]. *Relativity* was associated with ambiguous (non-interpretable) data. When ambiguity occurs, numerical procedures, such as statistical analyses and mathematical modeling, cannot be conducted [16]. Data ambiguity -here observed in infections- has also been reported in neurology [16].

To prevent ambiguity, a method was designed to reduce noise, detect complexity, and capture anti-microbial immune dynamics (temporal changes). To reduce variability, the data were structured as *single* (one data point-wide) *lines of data points*. This strategy was combined with the analysis of *complexity* and *temporal data directionality*. Because no uni- or bi-dimensional plot can reveal 3D/4D patterns, to detect complexity, the *circularity* of 3D data interactions was assessed [9, 10, 31]. Arrows that expressed where each data point came from/went to measured *temporal data flows*, even when they occurred within brief timeframes.

The use of arrows that connected pairs of consecutive observations met the definition proposed by Nielsen and Jorgensen: ‘orientors’ are indicators that identify *short-term*, *immediate directionality* (qualitative information) but lack long-term predictability [20]. Yet, the 4D method exceeded such criteria: it analyzed qualitative and quantitative data.

In agreement with earlier reports, distinct patterns emerged when, over time, one indicator changed in larger (or smaller) magnitudes than the remaining indicators [43]. Because the 4D method demonstrated multi-directional data flows, the central assumption of reductionist methods (the presence of only uni-directional flows) was rejected [22].

The impact of the 3D/4D combinatorial approach was summarized in the contrast shown by Figs 2d, 8a and 8b: while the *same data* were analyzed with the *same variables* (and 3D plots were used in both analyses), no discrimination (ambiguity) was revealed in Fig 2d, but distinct (and informative) patterns were conveyed by Fig 8a and 8b. The difference was due to a double strategy, implemented in Fig 8a and 8b: (i) the construction and measurement of interactions that involved two or more cell types (not isolated variables, such as the lymphocyte percent [measured in Fig 2d]), and (ii) the observation of perpendicular patterns–a procedure that separates subsets that differ in one or more immunological function(s) and, given their non-overlapping distributions, results in statistically significant differences (Fig 8b). This means that statistical analysis should be conducted after (not before) data subsets are distinguished [9].

### Clinical applications and extraction of new information

The 4D approach partitioned the data into subsets [44]. Applications include: (i) *earlier* detection of infection, (ii) identification of subsets that differ in *immune profiles* (pathogenesis-based *diagnostics*), (iii) *selection* and *evaluation of therapies*, and (iv) prevention of *ambiguity*.

For instance, canine leukocyte patterns detected inflammation even when bacteriological tests were negative (open circle with a cross embedded, Fig 4d). Such information enables clinicians to intervene and/or prognosticate, earlier.

Higher M/L values were observed in the MSSA recurrent infection, after antibiotics were prescribed for the first time (Fig 5c). Such profile was consistent with reports that indicate monocytes increase earlier (approximately 3 days after initiation of an immune response) than lymphocytes [45]. However, because high M/L values were also observed after cessation of antibiotic treatments (in recurrent infections), the 4D method may also be used to explore topics of major medical interest: *antibiotic-immunological-bacterial-temporal* interactions [46].

New (and *in vivo*) methods are needed to reduce lengthy procedures and other limitations associated with *in vitro* perspectives. For example, antibiotic susceptibility tests cannot measure tissue environmental conditions, such as hypoxia [47]. While classic methods have focused on the microbe and used *in vitro* (or, when animal models are used, *in vivo*) approaches, such methods are error-prone because they (i) do not assess poly-microbial infections, (ii) ignore the fact that animal models do not truly represent human infection conditions, (iii) do not account for differences across individuals, and (iv) do not measure immune dynamics [48–52].

Earlier and more informative methods are also needed to evaluate antibiotics [53, 54]. Such new methods could consider (i) the predominant cell type(s) involved in some infections and (ii) the fact that some antibiotics synergize with (or inhibit) specific cell types [55–58]. For instance, immune responses against *Salmonella* species differ: those against typhoid fever-causing agents are monocyte-mediated, while responses against gastroenteritis-causing serovars are neutrophil-mediated [58]. In *Pseudomonas aeruginosa-*related infections, amikacin is synergistic with neutrophils but ciprofloxacin is not [59]. In spite of such reports, no method is available to explore *antibiotic-immunologic-microbial-temporal* relationships with *earlier*, *in vivo* inputs.

Changes in directional flows (observed even within a few hours) demonstrated earlier evaluations of antibiotic therapy are feasible and data ambiguity may be prevented. For example, the *temporal flows* of human leukocyte data demonstrated (three times) that antimicrobial interventions may interact not only with the microbe but also with the immune system (days 160–161 [hip implant/MSSA case, Fig 5d], and days 119–120 and 135–146 [HIV case, Fig 6e and 6f]).

This evaluation–conducted as a part of a process aimed at exploring the properties of infectious disease data–may, later, be implemented by clinicians that use a clinician-friendly software package. While such a package has not yet been developed (and, therefore, this approach is not ready for application in clinical settings), a simpler but informative approximation is already feasible: when data points are located within the range characterized by high P/L and N/L, as well as low MC/N values, an infection cannot be ruled out (red symbols, Fig 8a); in contrast, when observations exhibit high MC/N and low P/L and N/L values, recovery is likely (blue symbols, Fig 8a). When such data subsets are perpendicular to one another, they tend to differ at statistical significant levels, as shown here (Tables I, J in S1 File). When *personalized* data are considered, the presence or absence of data inflections may support or modify earlier decisions. For instance, when a data inflection is observed within one day, it may be suspected that such change is not a random effect and, consequently, earlier (diagnostic- and/or treatment-related) decisions may be defensible (Fig 9a). In contrast, when temporal data flows do not change in directionality, it may be suspected that earlier decisions were not effective and, consequently, they might be reevaluated (Fig 9e). When only a minor change in the data flow is observed (Fig 9b), an additional test (conducted a few hours later) and/or pattern amplification strategies (e.g., reducing the scale of the axis of interest) may improve data visualization.

While no data representation can replace clinical expertise, data visualizations that integrate several leukocyte data combinations may facilitate earlier interpretations. When–after a therapy is prescribed in response to a neutrophil-predominant profile, i. e., one characterized by a high N/L value–a shift toward a mononuclear cell-predominant profile is observed, the hypothesis that the infection is progressing toward recovery is supported. In contrast, when a neutrophil-predominant profile remains even after therapy, a revision of the earlier decision may be considered. Because directionality-based analyses do not require population-based metrics, 4D methods may apply to *personalized* medicine–where new methods are needed [18, 44].

The 4D analysis of infectious disease-related data properties–including dynamics and complexity–can prevent ambiguity and foster data partitioning into subsets, facilitating earlier, personalized, explanatory (immunology-based) inferences. To further explore such properties, prospective studies are recommended.

## Supporting Information

S1 File Longitudinal leukocyte and microbial data analyzed in these studies. (DOC)
