# Supplementary material for: Preventing Data Ambiguity in Infectious Diseases with Four-Dimensional and Personalized Evaluations
Source: PLoS One. 2016 Jul 13;11(7):e0159001. doi: 10.1371/journal.pone.0159001 (PMC4943638; doi:10.1371/journal.pone.0159001)
Supplement: S1 File — (DOC) [file pone.0159001.s001.doc]

**Table A -Human (sepsis) leukocyte/microbial data and subset classification (n=7)**

| **Case**  **/Day** | **Microbial isolation** | **N %** | **L %** | **M %** | **Spatial subset*** | **Spatial-temporal subset*** |
| --- | --- | --- | --- | --- | --- | --- |
| 1.1 | *Escherichia coli* | 96.5 | 2.4 | 1.0 | Vertical | Vertical |
| 1.2 | *Escherichia coli* | 96.7 | 3.0 | 0.2 | Vertical | V-TD |
| 1.3 | *Escherichia coli* | 94.7 | 3.6 | 1.7 | Vertical | V-TD |
| 2.1 | *Escherichia coli* | 86.5 | 5.6 | 7.8 | Right | Right |
| 2.2 | *Escherichia coli* | 73.8 | 13.4 | 12.7 | Right | R-LTR |
| 2.3 | *Escherichia coli* | 65.9 | 16.9 | 17.1 | Right | R-LTR |
| 3.1 | *Enterococcus faecalis* | 73.6 | 18.2 | 8.1 | Right | Right |
| 3.2 | *Enterococcus faecalis* | 78.2 | 14.3 | 7.4 | Right | R-RTL |
| 3.3 | *Enterococcus faecalis* | 87.9 | 6.6 | 5.4 | Right | R-RTL |
| 4.1 | *Acinetobacter baumannii* | 87.8 | 10.8 | 1.3 | Vertical | Bottom |
| 4.2 | *Acinetobacter baumannii* | 92.2 | 7.6 | 0.1 | Vertical | B-RTL |
| 4.3 | *Acinetobacter baumannii* | 81.5 | 15.5 | 3.0 | Vertical | B-LTR |
| 5.1 | *Proteus mirabilis* | 87.8 | 8.4 | 3.7 | Vertical | Bottom |
| 5.2 | *Proteus mirabilis* | 81.7 | 13.5 | 4.7 | Right | B-LTR |
| 6.1 | *Enterococcus faecalis* | 88.1 | 10.0 | 1.8 | Vertical | Bottom |
| 6.2 | *Enterococcus faecalis* | 51.2 | 47.2 | 1.5 | Left | B-RTL |
| 7.1 | *S. liquefaciens* | 76.5 | 11.2 | 12.2 | Right | Right |
| 7.2 | *Serratia liquefaciens* | 94.4 | 2.9 | 2.6 | Vertical | B-RTL |
| 7.3 | *Serratia liquefaciens* | 92.2 | 3.4 | 4.3 | Right | V-TD |

- Subset identified according to spatial (3D) or spatial-temporal (4D) patterns:
- V-TD: vertical, top-down; R-LTR: right/left-to-right; R-RTL: right/right-to-left; B-RTL : bottom/right-to-left; B-LTR: bottom/left-to-right;

**Table B -HHuman (MSSA septic hip) leukocyte/microbial data and subset classification**

**Fig. 3**

| **Days#** | **Microbial isolation / clinical features and/or treatment** | **N %** | **L %** | **M %** | **Spatial subset*** | **Spatial-temporal subset*** |
| --- | --- | --- | --- | --- | --- | --- |
| D1 | High N/L | 84.8 | 10.1 | 5.0 | Right | Right |
| D2 | *MSSA*/septic hip | 88.7 | 5.1 | 6.1 | Right | Right |
| D3 | Same as above | 92.0 | 4.0 | 4.0 | Right | Right |
| D51 | Low N/L --Vancomycin | 65.9 | 23.4 | 10.6 | Bottom, left | Bottom, left |
| D79 | Rifampin/nafcillin | 67.7 | 20.8 | 11.4 | Bottom, left | Bottom, left |
| D107 | Rifampin/nafcillin | 63.0 | 26.1 | 10.9 | Bottom, left | Bottom, left |
| D128 | Discharge – no antibiotic | 70.8 | 20.2 | 8.9 | Bottom, left | Bottom, left |
| D158 | Recurrence--no antibiotic | 73.6 | 13.7 | 12.6 | Vertical | V-BU |
| D159 | Cefazolin/rifampin | 73.2 | 12.4 | 14.4 | Vertical | V-BU |
| D160 | Cefazolin | 72.9 | 11.5 | 15.6 | Vertical | V-BU |
| D161 | Cefazolin--flow changes | 69.8 | 15.6 | 14.6 | Vertical | V-TD |
| D164 | Cefazolin | 67.4 | 18.9 | 13.7 | Vertical | V-TD |
| D191 | Rifampin | 72.9 | 16.7 | 10.4 | Bottom, left | Bottom, left |
| D223 | Nafcillin | 72.2 | 18.6 | 9.3 | Bottom, left | Bottom, left |
|  |  |  |  |  |  |  |

* Subset identified according to spatial (3D) or spatial-temporal (4D) patterns: (i) V-BU: vertical, bottom-up; (ii) V-TD: vertical, top-down.

#: Time is expressed as consecutive days (‘day 1’ is the date when the first visit took place).

**Table C -HHuman leukocyte/microbial data and subset classification (HIV/MRSA)**

| **Days#** | **Microbial isolation / clinical features and/or treatment** | **N %** | **L %** | **M %** | **Spatial subset*** | **Spatial-temporal subset*** |
| --- | --- | --- | --- | --- | --- | --- |
| D13 | No antibiotics | 58.7 | 26.2 | 15.0 | RH | RH |
| D68 | No antibiotics | 53.0 | 21.2 | 25.7 | RH | RH |
| D69 | Low N/L- No antibiotics Other | 36.5 | 48.7 | 14.6 | RH | RH |
| D72 | No antibiotics | 24.5 | 70.1 | 5.2 | RH | RH |
| D73 | No antibiotics | 35.7 | 42.8 | 21.4 | RH | RH |
| D74 | Toxoplasmosis -- No antibiotics | 37.1 | 41.4 | 21.4 | RH | RH |
| D75 | No antibiotics | 28.3 | 55.5 | 16.0 | RH | RH |
| D76 | No antibiotics | 50.0 | 36.4 | 13.5 | RH | RH |
| D84 | Flow changes | 56.1 | 32.5 | 11.2 | LH | LH |
| D92 | Fever, *MSSA*--Vancomycin starts | 69.6 | 22.2 | 8.0 | LH | LH |
| D95 | Flow changes --Vancomycin | 57.0 | 33.0 | 10.0 | LH | LH |
| D96 | Vancomycin | 61.6 | 31.3 | 7.0 | LH | LH |
| D111 | Vancomycin | 68.4 | 18.4 | 13.1 | LH | LH-BU |
| D118 | N/L increases --Vancomycin | 88.1 | 6.4 | 5.3 | TV | TV |
| D119 | N/L decreases --Vancomycin | 76.0 | 14.1 | 9.7 | LH | LH-TD |
| D126 | Skin rash --Vancomycin stops | * | * | * | * | * |
| D127 | No antibiotic treatment | 70.5 | 15.3 | 14.1 | LH | LH |
| D134 | *MRSA* --Vancomycin re-starts | 72.0 | 20.0 | 8.0 | LH | LH-BU |
| D135 | N/L increases --Vancomycin | 87.5 | 8.3 | 4.1 | TV | TV |
| D136 | N/L decreases --Vancomycin | 76.9 | 14.1 | 8.9 | LH | LH-TD |
| D138 | Vancomycin | 65.3 | 26.6 | 8.0 | LH | LH |

**Fig. 3**

* Subset identified according to spatial (3D) or spatial-temporal (4D) patterns: (i) RH: right horizontal; (ii) LH: left horizontal; (iii) LH-BU: left horizontal, bottom-up; (iv) TV: top vertical; (v)

LH-TD: left horizontal, top-down.

**Table D -Canine leukocyte/microbial data and subset classification**

**Fig. 3**

| **Time (date)** | **Microbial isolation** | **N %** | **L %** | **M %** | **Spatial subset*** | **Spatial-temporal subset*** |
| --- | --- | --- | --- | --- | --- | --- |
| 5/11/2012 | negative | 57.9 | 35.2 | 6.8 | Left | Start |
| 11/2/2012 | negative | 64.4 | 28.1 | 7.4 | Left | LS/RF |
| 11/26/2012 | negative | 63.6 | 29.7 | 6.6 | Left | LS/LF |
| 11/29/2012 | *Enterobacter cloacae* | 89.1 | 7.1 | 3.6 | Right | RS/RF |
| 12/7/2012 | negative | 83.6 | 10.2 | 6.1 | Right | RS/LF |
| 12/31/2012 | negative | 74.7 | 19.1 | 6.1 | Left | LS/LF |
| 1/19/2013 | *Staphylococcus pseudointermedius* | 85.7 | 9.1 | 5.1 | Right | RS/RF |
| 1/28/2013 | negative | 76.0 | 19.8 | 4.0 | Left | LS/LF |
| 2/26/2013 | negative | 73.4 | 20.5 | 6.0 | Left | LS/TD |
| 3/21/2013 | negative | 88.0 | 7.6 | 4.3 | Right | RS/RF |
| 4/10/2013 | negative | 74.4 | 21.4 | 4.1 | Left | LS/LF |

* Subset identified according to spatial (3D) or spatial-temporal (4D) patterns: (i) LS/RF: left side/right flow; (ii) LS/LF: left side/left flow; (iii) RS/RF: right side/right flow; (iv) RS/LF: right side/left flow; (v) LS/TD: left side/top-down flow.

**Table E -Statistical comparisons between canine (spatial and spatial-temporal) subsets described in Table D.**

| **Canine study --Spatial subsets** | *P* value (Mann-Whitney test for the median) |
| --- | --- |
| N % (left subset, n=7) vs. N %8 (right subset, n=4) | **< 0.02** |
| L % (left subset, n=7) vs. L % (right subset, n=4) | **< 0.02** |
| M % (left subset, n=7) vs. M % (right subset, n=4) | >0.10 |
| N/L (left subset, n=7) vs. N/L (right subset, n=4) | **< 0.02** |
| M/N (left subset, n=7) vs. M/N (right subset, n=4) | =0.07 |
| M/L (left subset, n=7) vs. M/L (right subset, n=4) | **< 0.02** |
|  |  |
| **Canine study –Spatial-temporal subsets** **(4D analysis)** | |
| N % (left-side/left flow, n=4) vs. N % (right-side/right flow, n=3) | = 0.05 |
| L % (left-side/left flow, n=4) vs. L % (right-side/right flow, n=3) | = 0.05 |
| M % (left-side/left flow, n=4) vs. M % (right-side/right flow, n=3) | >0.10 |
| N/L (left-side/left flow, n=4) vs. N/L (right-side/right flow, n=3) | = 0.05 |
| M/N (left-side/left flow, n=4) vs. M/N (right-side/right flow, n=3) | >0.10 |
| M/L (left-side/left flow, n=4) vs. M/L (right-side/right flow, n=3) | = 0.05 |

- Only subsets that achieved two or more statistically significant differences are reported.

**Table F -Statistical comparisons between human MSSA/septic hip (spatial and spatial-temporal) subsets described in Table B.**

| **Human MSSA/septic hip--Spatial subsets** | *P* value (Mann-Whitney test for the median) |
| --- | --- |
| N % (right, n=3) vs. N % (bottom left, n=6) | **< 0.03** |
| L % (right, n=3) vs. L % (bottom left, n=6) | **< 0.03** |
| M % (right, n=3) vs. M % (bottom left, n=6) | **< 0.03** |
| N/L (right, n=3) vs. N/L (bottom left, n=6) | **< 0.03** |
| M/N (right, n=3) vs. M/N (bottom left, n=6) | **< 0.03** |
| M/L (right, n=3) vs. M/L (bottom left, n=6) | >0.10 |
|  |  |
| N % (right, n=3) vs. N % (vertical, n=5) | **< 0.04** |
| L % (right, n=3) vs. L % (vertical, n=5) | **< 0.04** |
| M % (right, n=3) vs. M % (vertical, n=5) | **< 0.04** |
| N/L (right, n=3) vs. N/L (vertical, n=5) | **< 0.04** |
| M/N (right, n=3) vs. M/N (vertical, n=5) | **< 0.04** |
| M/L (right, n=3) vs. M/L (vertical, n=5) | >0.10 |
|  |  |
| N % (bottom left, n=6) vs. N % (vertical, n=5) | >0.10 |
| L % (bottom left, n=6) vs. L % (vertical, n=5) | **< 0.03** |
| M % (bottom left, n=6) vs. M % (vertical, n=5) | **< 0.01** |
| N/L (bottom left, n=6) vs. N/L (vertical, n=5) | **< 0.03** |
| M/N (bottom left, n=6) vs. M/N (vertical, n=5) | **< 0.02** |
| M/L (bottom left, n=6) vs. M/L (vertical, n=5) | **< 0.01** |
|  |  |
| **Human MSSA/septic hip** **–Spatial-temporal subsets** **(4D analysis)** | |
| N % (vertical/bottom-up, n=3) vs. N % (bottom left, n=6) | **< 0.04** |
| L % (vertical/bottom-up, n=3) vs. L % (bottom left, n=6) | **< 0.03** |
| M % (vertical/bottom-up, n=3) vs. M % (bottom left, n=6) | **< 0.03** |
| N/L (vertical/bottom-up, n=3) vs. N/L (bottom left, n=6) | **< 0.03** |
| M/N (vertical/bottom-up, n=3) vs. M/N (bottom left, n=6) | =0.05 |
| M/L (vertical/bottom-up, n=3) vs. M/L (bottom left, n=6) | **< 0.03** |
|  |  |

**Table G -Statistical comparisons between human MRSA/HIV (spatial and spatial-temporal) subsets described in Table C.**

| **Human case II (MRSA**/**HIV**)-**Spatial subsets** | *P* value (Mann-Whitney test for the median) |
| --- | --- |
| N % left horizontal (n=10) vs. N % right horizontal (n=8) | **<0.01** |
| L % left horizontal (n=10) vs. L % right horizontal (n=8) | **<0.01** |
| M % left horizontal (n=10) vs. M % right horizontal (n=8) | **<0.02** |
| N/L left horizontal (n=10) vs. N/L right horizontal (n=8) | **<0.01** |
| M/N left horizontal (n=10) vs. M/N right horizontal (n=8) | **<0.01** |
| M/L left horizontal (n=10) vs. M/L right horizontal (n=8) | <0.05 |
|  | |
| **Human case II (MRSA**/**HIV**)-**Spatial-temporal subsets** |  |
| N % left horizontal (n=6) vs. N % right horizontal (n=8) | **<0.01** |
| L % left horizontal (n=6) vs. L % right horizontal (n=8) | =0.06 |
| M % left horizontal (n=6) vs. M % right horizontal (n=8) | **<0.04** |
| N/L left horizontal (n=6) vs. N/L right horizontal (n=8) | **<0.04** |
| M/N left horizontal (n=6) vs. M/N right horizontal (n=8) | **<0.01** |
| M/L left horizontal (n=6) vs. M/L right horizontal (n=8) | <0.05 |
| N % top vertical (n=2) vs. N % right horizontal (n=8) | =0.05 |
| L % top vertical (n=2) vs. L % right horizontal (n=8) | =0.05 |
| M % top vertical (n=2) vs. M % right horizontal (n=8) | =0.09 |
| N/L top vertical (n=2) vs. N/L right horizontal (n=8) | =0.05 |
| M/N top vertical (n=2) vs. M/N right horizontal (n=8) | =0.05 |
| M/L top vertical (n=2) vs. M/L right horizontal (n=8) | >0.10 |
|  | |

**Table H -Statistical comparisons between human septic (spatial) subsets described in Table A.**

| **Human sepsis (3D [T/BAR/AAW) -Spatial analysis** | *P* value (Mann-Whitney test for the median) |
| --- | --- |
| N % (right, n=9) vs. N % (vertical, n=9) | **< 0.01** |
| L % (right, n=9) vs. L % (vertical, n=9) | =0.08 |
| M % (right, n=9) vs. M % (vertical, n=9) | **< 0.01** |
| N/L (right, n=9) vs. N/L (vertical, n=9) | = 0.08 |
| M/N (right, n=9) vs. M/N (vertical, n=9) | **< 0.01** |
| M/L (right, n=9) vs. M/L (vertical, n=9) | **< 0.01** |
|  | |
| **Human sepsis (3D [T/BAR/AAW) –Spatial-temporal analysis** |  |
| N % (vertical, top-down, n=3) vs. N % (right, n=3) | =0.08 |
| L % (vertical, top-down, n=3) vs. L % (right, n=3) | =0.08 |
| M % (vertical, top-down, n=3) vs. M % (right, n=3) | =0.08 |
| N/L (vertical, top-down, n=3) vs. N/L (right, n=3) | =0.08 |
| M/N (vertical, top-down, n=3) vs. M/N (right, n=3) | =0.08 |
| M/L (vertical, top-down, n=3) vs. M/L (right, n=3) | >0.10 |

**Table I -Human (sepsis) leukocyte spatial subset classification based on low-complexity indicators (interactions between two or more cell types, n=7)**

| Case ID | Day | L% | N% | M% | P % | MC % | P/L | MC/N | N/L | Spatial subset |
| --- | --- | --- | --- | --- | --- | --- | --- | --- | --- | --- |
| 1 | 1 | 2.45 | 96.55 | 1.00 | 97.55 | 3.45 | 39.81 | 0.03 | 39.40 | B |
| 1 | 2 | 3.05 | 96.70 | 0.25 | 96.95 | 3.30 | 31.78 | 0.03 | 31.70 | B |
| 1 | 3 | 3.60 | 94.70 | 1.70 | 96.40 | 5.30 | 26.77 | 0.05 | 26.30 | B |
| 2 | 1 | 5.60 | 86.55 | 7.85 | 94.40 | 13.45 | 16.85 | 0.15 | 15.45 | B |
| 2 | 2 | 13.45 | 73.85 | 12.70 | 86.55 | 26.15 | 6.43 | 0.35 | 5.49 | A |
| 2 | 3 | 16.96 | 65.94 | 17.10 | 83.04 | 34.06 | 4.89 | 0.51 | 3.88 | A |
| 3 | 1 | 18.25 | 73.65 | 8.10 | 81.75 | 26.35 | 4.47 | 0.35 | 4.03 | A |
| 3 | 2 | 14.35 | 78.25 | 7.40 | 85.65 | 21.75 | 5.96 | 0.27 | 5.45 | A |
| 3 | 3 | 6.65 | 87.90 | 5.45 | 93.35 | 12.10 | 14.03 | 0.13 | 13.21 | B |
| 4 | 1 | 10.85 | 87.80 | 1.35 | 89.15 | 12.20 | 8.21 | 0.13 | 8.09 | B |
| 4 | 2 | 7.65 | 92.25 | 0.10 | 92.35 | 7.75 | 12.07 | 0.08 | 12.05 | B |
| 4 | 3 | 15.50 | 81.50 | 3.00 | 84.50 | 18.50 | 5.45 | 0.22 | 5.25 | A |
| 5 | 1 | 8.45 | 87.85 | 3.70 | 91.55 | 12.15 | 10.83 | 0.13 | 10.39 | B |
| 5 | 2 | 13.55 | 81.75 | 4.70 | 86.45 | 18.25 | 6.38 | 0.22 | 6.03 | A |
| 6 | 1 | 10.00 | 88.15 | 1.85 | 90.00 | 11.85 | 9.00 | 0.13 | 8.81 | B |
| 6 | 2 | 47.25 | 51.25 | 1.50 | 52.75 | 48.75 | 1.11 | 0.95 | 1.08 | A |
| 7 | 1 | 11.25 | 76.55 | 12.20 | 88.75 | 23.45 | 7.88 | 0.30 | 6.80 | A |
| 7 | 2 | 2.95 | 94.45 | 2.60 | 97.05 | 5.55 | 32.89 | 0.05 | 32.01 | B |
| 7 | 3 | 3.45 | 92.25 | 4.30 | 96.55 | 7.75 | 27.98 | 0.08 | 26.73 | B |

P: phagocyte (neutrophil and monocyte)

MC: mononuclear cell (lymphocyte and monocyte)

P/L: phagocyte/lymphocyte

N/L: neutrophil/lymphocyte

**Table J Statistical comparisons between human septic (spatial) subsets defined by low-complexity indicators (phagocyte/lymphocyte [P/L], mononuclear cell/neutrophil [MC/N], and neutrophil/lymphocyte [N/L] ratios).**

| **Human sepsis –Spatial analysis** | *P* value (Mann-Whitney test for the median) |
| --- | --- |
| Lymphocyte % (set A, n=8, vs. set B, n=11) | **<0.001** |
| Neutrophil % (set A, n=8, vs. set B, n=11) | **<0.001** |
| Monocyte % (set A, n=8, vs. set B, n=11) | **<0.02** |
| Phagocyte % (set A, n=8, vs. set B, n=11) | **<0.001** |
| Mononuclear cell % (set A, n=8, vs. set B, n=11) | **<0.001** |
| Phagocyte/lymphocyte ratio (set A, n=8, vs. set B, n=11) | **<0.001** |
| Neutrophil/lymphocyte ratio (set A, n=8, vs. set B, n=11) | **<0.001** |
| Mononuclear cell/neutrophil (set A, n=8, vs. set B, n=11) | **<0.001** |

- No statistical analysis of the spatial-temporal patterns is conducted because a personalized analysis was implemented according to non-numerical (directionality-based) temporal data flows, as shown in Fig. 9.

The following instructions allow readers to reproduce plots and analyses related to the **Table I dataset**.

- - - By plotting the continuous data provided in the **Table I dataset** while considering the

discontinuous spatial patterns detected in Fig. 8a (‘subset A’, ‘subset B’), the plot reported in Fig. 8B can be reproduced.

- - - Fig. 8B demonstrates that blood leukocyte data possess hidden interactions

(not observed in non-structured data, as reported in Fig. 1), which may be uncovered when 3D patterns and low-complexity indicators are analyzed.

- - - After data subsets are differentiated according to spatial patterns (sets A and B),

comparisons between subsets will demonstrate numerous immunological differences

which –given the non-overlapping distributions of the two data subsets (Fig. 8A)– will

also achieve statistical significance, as shown above.

- - - In addition, personalized assessments can be conducted on daily basis, as reported in Fig. 9.
